# Ensemble of Time-Evolving SASP Gene Sets Identifies IGFBP7 and CDKN1A as a Potential Marker Pair for Senescent Fibroblast Subpopulations Across Tissues

**DOI:** 10.3390/ijms27073012

**Published:** 2026-03-26

**Authors:** Hyunsoo Kim, Erich Kummerfeld, Laura J. Niedernhofer, Constantin Aliferis, Paul D. Robbins, Jinhua Wang

**Affiliations:** 1 Institute for Health Informatics, University of Minnesota Twin Cities, Minneapolis, MN 55455, USA; kimx0662@umn.edu (H.K.);; 2 Masonic Cancer Center, University of Minnesota Twin Cities, Minneapolis, MN 55455, USA; 3 Masonic Institute on the Biology of Aging and Metabolism, Department of Biochemistry, Molecular Biology and Biophysics, University of Minnesota Twin Cities, Minneapolis, MN 55455, USA

**Keywords:** single-cell RNA sequencing, cellular senescence, temporal dynamics of SASP, fibroblast subpopulations, IGFBP7, CDKN1A (p21), gene-set scoring, SASP score/EGS

## Abstract

The senescence-associated secretory phenotype (SASP) is a hallmark of senescent cells and plays a critical role in the development and progression of various age-related diseases, including cancer, cardiovascular disorders, and neurodegenerative diseases. In this study, we characterize SASP heterogeneity using single-cell RNA sequencing (scRNA-seq) data, focusing on the transcriptional signatures associated with elevated expression of individual SASP genes in mature senescent cells, as well as time-dependent variation in SASP expression across the early and mature senescent states in the WI-38 human lung fibroblast cell line. We generated multiple gene sets, each representing the transcriptional landscape linked to high expression of a specific SASP gene, and integrated them into an ensemble that reflects the temporal dynamics of SASP gene expression. Applying SASP scores derived from this ensemble of gene sets (SASP scores/EGS) to publicly available scRNA-seq datasets from human lung, skin, and eye tissues enabled the identification of senescent fibroblasts and revealed *IGFBP7* as a consistently upregulated marker in p21^+^ or p16^+^ fibroblasts across diverse human tissues. Our framework supports improved detection of both early and mature fibroblast replicative senescent cells, offering valuable insights into aging and age-related disease research.

## 1. Introduction

Cellular senescence is a significant biological process intricately associated with several critical facets of biology and human health. It serves as a vital component in maintaining normal tissue homeostasis by acting as a check on excessive cell proliferation. Furthermore, it is intriguingly linked to aging, given its tendency to accumulate within tissues over time and contribute to age-related diseases. In embryogenesis, senescence takes on a pivotal role in tissue remodeling and differentiation during embryonic development. It also contributes to wound healing through tissue remodeling. Importantly, senescence can be triggered by a variety of factors, including chemotherapy, oncogene activation, and cellular stresses that induce DNA damage, as well as numerous other senescence-inducing stimuli that continue to be uncovered. In particular, biomarkers such as SA-β-gal, p21, CCL2, and IGFBP3 have been identified for therapy-induced senescence in breast cancer [1].

Recent advancements have seen the emergence of multiple gene sets associated with senescence, exemplified by a senescence-related gene set of SenMayo [2]. The characteristics of cellular senescence include dysfunctional mitochondria [3], lysosomal dysfunction [4], ER stress [5], resistance to apoptosis [6], increased cell size [7], loss of lamin B1 [8], the disruption of nuclear envelope integrity [8], and the senescence-associated secretory phenotype (SASP) [9]. Secreted proteins from senescent cells can influence neighboring normal or tumor cells. Senescent cells can be characterized by their distinct patterns of SASP gene expression, and the expression levels of a SASP gene across cells might be associated with those of other SASP genes and other non-SASP genes.

In this study, we developed an ensemble of gene sets consisting of both upregulated and downregulated genes specific to subpopulations of senescent cells in which a SASP gene or another gene of interest was highly expressed by comparing these senescent cells with non-senescent cells. These senescence-related gene signatures, derived from temporal SASP transcriptional heterogeneity, can be used to predict the presence of senescent cells in single-cell RNA-seq (scRNA-seq) datasets obtained from human tissue samples.

## 2. Results

### 2.1. Heterogeneity of SASP Factors

**Figure 1:**
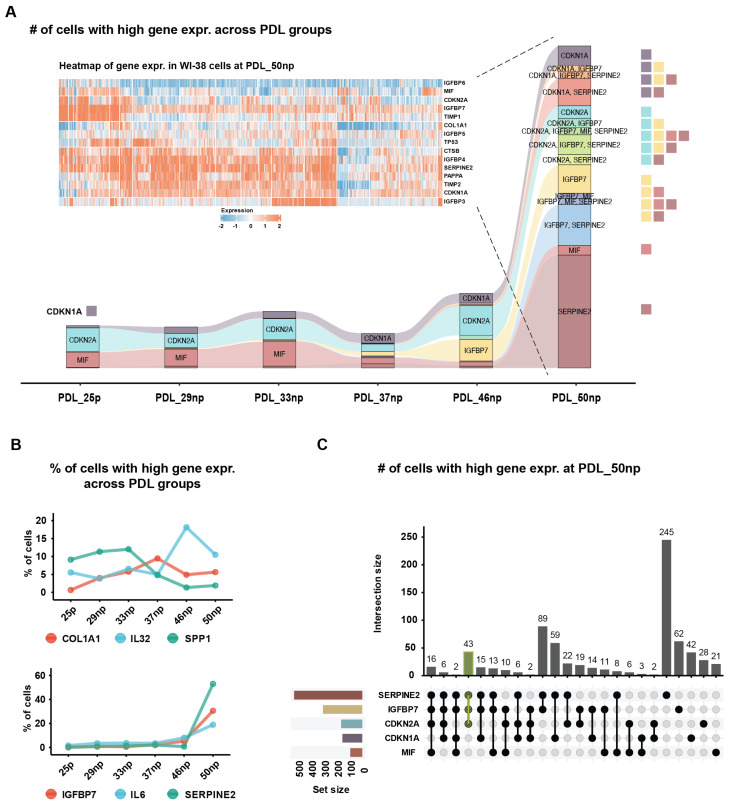
Transcriptional heterogeneity of SASP genes and other genes of interest in the WI-38 human lung fibroblast cell line. (**A**) Flow diagram across temporal PDL groups shows the number of cells with high gene expression (z-score > 1.5) for five selected genes (i.e., *CDKN1A* (p21), *CDKN2A* (p16), *MIF*, *IGFBP7*, and *SERPINE2*). The heatmap displays the gene expression in WI-38 cells at PDL_50np of the five genes and some additional genes of interest (i.e., *IGFBP3*, *IGFBP4*, *IGFBP5*, *IGFBP6*, *TIMP1*, *TIMP2*, *COL1A1*, *CTSB*, *PAPPA*). (**B**) Percentage of cells with high SASP gene expression across PDL groups. (**C**) Number of cells with high gene expression and co-expression at PDL_50np, along with dotted lines indicating the intersection of sets when considering the five genes. For example, there were 43 cells where *CDKN2A*, *IGFBP7*, and *SERPINE2* were highly expressed.

It has been well established that SASP factors released by senescent fibroblasts can impact cancer cells in several ways: (1) cancer cell proliferation via IL6, IL8, AREG, CCL5, CXCL12, OPN, HGF, and MMPs; (2) cancer cell invasion and migration via MMPs and chemerin (*RARRES2*); (3) cancer cell angiogenesis via VEGF and CTGF; and (4) protection of cancer cells from chemotherapy via sFRP2 and WNT16B [10]. Our analysis of scRNA-seq data obtained from the WI-38 human lung fibroblast cell line [11] revealed that not all senescent fibroblasts transcribe all SASP genes. Instead, these genes were primarily expressed in a subpopulation of early or mature senescent fibroblasts (Figure 1 and Figure S2).

To investigate the transcriptional diversity of SASP gene expression during cellular senescence, we analyzed scRNA-seq data from WI-38 human lung fibroblasts across multiple population doubling levels (PDLs). We observed that only subsets of cells within each senescent stage exhibited elevated expression of canonical SASP genes, indicating substantial heterogeneity. For instance, in mature senescent cells (PDL_50np), genes such as *CDKN2A*, *IGFBP7*, and *SERPINE2* were co-expressed in a specific subset of cells, suggesting the existence of distinct senescent subpopulations. This pattern was visualized by quantifying the number and proportion of cells exhibiting high expression (z-score > 1.5) of selected SASP and senescence-associated genes across PDLs. It is worth noting that overlap in gene expression among these markers revealed that senescent cells do not uniformly express all SASP components but rather display combinatorial expression patterns. These findings, summarized in Figure 1, underscore the complexity of SASP regulation and support the notion that senescence is not a uniform state but comprises multiple transcriptionally distinct subtypes.

Here, we describe examples of SASP gene expression heterogeneity in WI-38 cells within the mature senescent cells (PDL_50np), as shown in Figure S2. *IL6* was highly expressed (z-score > 1.5) only in a subset of cells. *HGF* was also highly expressed in certain cells. *MMP1* and *MMP2* were generally expressed, but not uniformly across all cells, whereas *MMP3* was highly expressed in a specific subpopulation in WI-38 cells at PDL_50np (cell cluster 24). The widespread expression of *MMP1* and *MMP2* supports the hypothesis that mature senescent cells may be linked to cancer cell invasion and migration when senescent cells are proximal to cancer cells. *VEGFA* was generally expressed in cell clusters 22 and 23 but showed low expression in other cell clusters at PDL_50np. *WNT16* was highly expressed in only a subpopulation of mature senescent cells. The SASP gene expression heterogeneity suggests that different subsets of senescent cells may play distinct roles against neighboring cells, including cancer cells.

Focusing on the earlier senescent cells at PDL_46np, we identified an additional layer of SASP heterogeneity: time-dependent SASP heterogeneity, which reflects differences in SASP gene expression between early and mature senescent cells. *IL32* was highly expressed in a subpopulation of PDL_46np (cell cluster 8). *CSF1* was highly expressed in a subpopulation of PDL_46np (cell cluster 10), although it was also detected at lower levels in some mature senescent cells.

SASP gene expression across PDL groups was summarized in a dot plot in Figure S2, where dot size represents the percentage of cells expressing each gene (nonzero expression) within a group. The highly expressed genes (yellow color) with smaller-sized dots clearly indicated that these genes were not universally expressed among all cells but rather in a subpopulation of cells within a PDL group. For instance, *CCL2* was only highly expressed in a subpopulation of WI-38 cells at PDL_50np. Many known SASP genes showed either no detectable expression or only very small dot sizes, indicating their absence or extremely weak expression in WI-38 fibroblasts and highlighting the cell-type dependency of SASP gene expression. *IGFBP5* generally showed higher expression in PDL_46np cells compared to PDL_50np. Some SASP genes (e.g., *DKK1*, *IL11*, and *VEGFC)* were also expressed in PDL_25p cells (Figure S3). Although this dot plot provided a simple and clear summary of gene expression within each PDL group, its dot size only represented the number of cells with nonzero expression rather than other thresholds.

The SASP gene expression heterogeneity observed in mature senescent cells, together with the time-dependent variability in SASP expression, motivated us to construct an ensemble of gene sets consisting of upregulated genes and downregulated genes identified in cells with high expression of a given SASP gene (z-score > 1.5). This ensemble of gene sets was used to predict senescent cells in WI-38 human lung fibroblasts as a cross-validation, aimed at demonstrating the usability of the ensemble of gene sets and measuring the confidence in their soundness. Further details on the method for predicting senescent cells using the ensemble of gene sets can be found in Section 4.

### 2.2. Prediction of Senescent Fibroblasts in Lung Tissue

**Figure 2:**
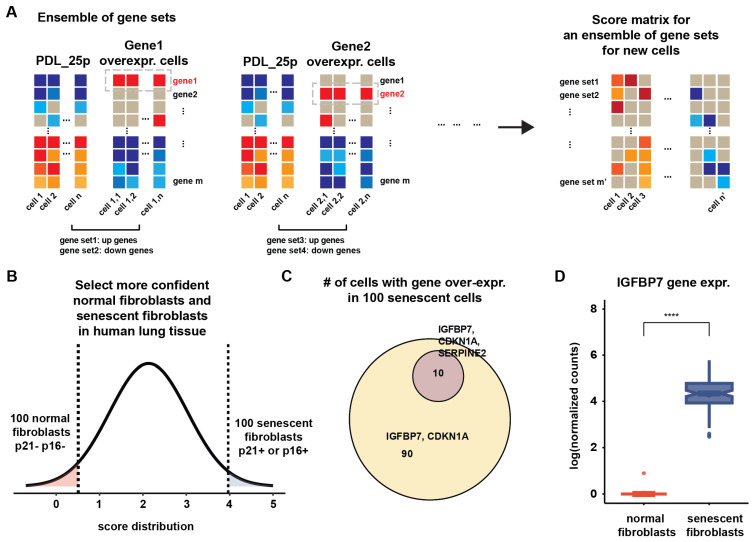
Construction and application of an ensemble of gene sets to identify senescent cells in human tissues. (**A**) Flow diagram outlines the process of constructing the ensemble gene sets. Upregulated and downregulated gene sets for each gene of interest, including SASP genes, were obtained by comparing cells in which each gene was overexpressed (z-score > 1.5) against control cells from the PDL_25p group. Lung fibroblast senescence scores for each gene set were calculated as the mean z-score of upregulated genes minus the mean z-score of downregulated genes. Z-scores were computed using the mean and standard deviation of gene expression in PDL_25p cells, which were stored during the earlier stage of ensemble gene set construction. Color scale represents gene expression levels, ranging from dark blue (lowest expression) to dark red (highest expression). (**B**) To identify more confidently classified normal and senescent fibroblasts in human lung tissue, we selected 100 cells from each group with the lowest and highest senescence scores, respectively. (**C**) Euler diagram illustrates the co-expression patterns of *CDKN1A*, *IGFBP7*, and *SERPINE2* in 100 senescent/stressed fibroblasts in lung tissue. (**D**) Log-normalized expression values of *IGFBP7* are shown for two groups: control (100 normal fibroblasts) and senescent (100 senescent/stressed fibroblasts) in lung tissue. The median expression levels between the two groups were significantly different (Wilcoxon rank-sum test, **** *p* < 1 × 10^−9^).

To predict senescent cells in lung fibroblast tissue, we used scRNA-seq lung data collected from the DISCO database [12]. Our ensemble of gene sets was derived from scRNA-seq data generated from the WI-38 human fibroblast cell line. The first step was to evaluate the utility of our ensemble of gene sets for predicting senescent fibroblasts in human lung tissue. We computed lung fibroblast senescence scores for all fibroblasts in the lung tissue dataset and identified the most confident normal and senescent fibroblasts by selecting the 100 p21^−^/p16^−^ cells with the lowest scores and the 100 p21^+^ or p16^+^ cells with the highest scores (see Figure 2 and Figure S4, and Section 4). The 100 predicted senescent cells represent <1% of 11,889 fibroblasts in human lung tissue.

There were similarities and differences in SASP gene expression between WI-38 cells and fibroblasts in human lung tissue. *IGFBP7* was highly expressed in senescent/stressed fibroblasts in lung tissue (Figure 2C,D). Although *IGFBP7* expression is strongly associated with replicative senescence (Figure 1A), it is also possible that other stressed cells may upregulate *IGFBP7*. For example, DNA damage activates TGF-β signaling [13] and TGF-β contributes to the induction of cellular senescence; senescent cells, in turn, secrete TGF-β as part of the SASP. Moreover, TGF-β participates in multiple signaling pathways and has broad, context-dependent effects [14]. Persistent TGF-β signaling drives sustained fibroblast activation [15], and lung fibroblasts in idiopathic pulmonary fibrosis (IPF) are known to develop senescent phenotypes [16]. Thus, *IGFBP7* alone may not be sufficient to identify senescent cells. To address this, our algorithm computes SASP scores by evaluating p21^−^/p16^−^ cells with the lowest scores and p21^+^ or p16^+^ cells with the highest scores.

TIMP1 was also highly expressed in senescent lung fibroblasts, although it was also elevated in some normal fibroblasts in lung tissue. In WI-38 cells, *IGFBP7* tended to be co-expressed with *CTSB*, *IGFBP4*, and *SERPINE2*. In contrast, in lung tissue, only part of this co-expression pattern, specifically *IGFBP7* together with *TIMP1*, *IGFBP6*, and *CDKN1A*, was observed (Figure S4A). After identifying the most confident p21^−^/p16^−^ control cells and p21^+^ or p16^+^ senescent cells, we identified differentially expressed genes that distinguished control and senescent fibroblasts in lung tissue (see Table S1, also downloadable from https://github.com/hyunsoo77/SASP_score, accessed on 10 March 2026).

### 2.3. Prediction of Senescent Fibroblasts in Other Tissues

To predict senescent fibroblasts in human skin tissue and human eye tissue, we utilized scRNA-seq fibroblast data collected by the DISCO database [12]. The next step was to apply our ensemble of gene sets obtained from a cell line to other tissues. This section differs from the previous one, which compared a lung cell line with lung tissue. Here, we test whether our method can predict senescent or stressed fibroblasts in other tissues, where differences arise not only from cell line versus tissue comparisons but also from variations in tissue location.

The prediction of senescent/stressed fibroblasts in human skin tissue was performed by computing lung fibroblast senescence scores for all fibroblast cells in skin tissue and selecting the most confident 100 normal skin fibroblasts and 100 senescent skin fibroblasts. The senescent skin fibroblasts exhibited high expression of *IGFBP7*, *CDKN1A*, *CXCL12*, *TIMP1/2*, and *IGFBP5*. *CCL2* was highly expressed in only some cells within senescent fibroblasts in skin tissue, which is consistent with its expression pattern in a cluster found in WI-38 cells at PDL_50np (Figure S5).

**Figure 3:**
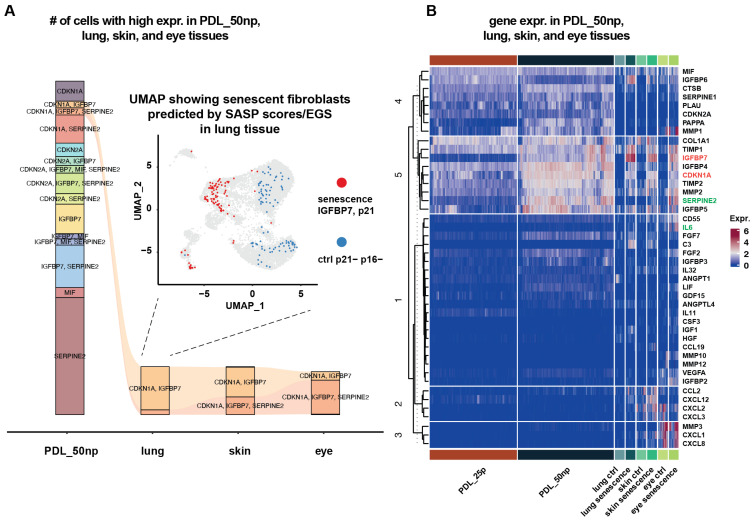
Heterogeneity of gene expression in SASP genes and other genes of interest in various human tissue fibroblasts. (**A**) A flow diagram illustrates the size of groups in WI-38 cells at PDL_50np, as well as fibroblasts in human lung, skin, and eye tissues, where the size of gene groups for five selected genes (i.e., *CDKN1A* (p21), *CDKN2A* (p16), *MIF*, *IGFBP7*, and *SERPINE2*) is the number of cells with high gene expression (z-score > 1.5) for a gene or for co-expression of multiple genes. This panel also includes a UMAP visualization of senescent p21^+^ fibroblasts predicted by SASP scores/EGS in lung tissue. (**B**) Log-normalized counts of gene expression are displayed for selected SASP genes, such as *IGFBP7* and *MMP1*, in normal fibroblasts in WI-38 (PDL_25p) and mature senescent fibroblasts in WI-38 (PDL_50np), as well as in control (normal) fibroblasts and senescent/stressed fibroblasts from human lung, skin, and eye tissues. Red font color in gene names indicates the marker pair *IGFBP7* and *CDKN1A*, and green font color highlights the genes of interest mentioned in the main text.

*IGFBP6* showed a relatively low expression in mature senescent WI-38 cells, whereas it was highly expressed in some cells in senescent fibroblasts in lung and skin tissues. This represents another level of SASP heterogeneity, specifically tissue-type SASP heterogeneity (Figure S5 and Figure 3B). Interestingly, the expression level of the *IGFBP6* gene in PDL_50np cells was found to be lower than in PDL25p cells, while the expression levels of other IGFBP genes were observed to be higher in PDL_50np cells.

The prediction of senescent/stressed fibroblasts in human eye tissue yielded the most confident 100 normal eye fibroblasts and 100 senescent eye fibroblasts. The senescent eye fibroblasts exhibited high expression of *IGFBP7*, *IGFBP2/4/5/6*, *SERPINE2*, *TIMP2*, *CD55*, *MIF*, and *CDKN1A* (Figure S6).

*MMP1* and *MMP3* were relatively highly expressed in mature senescent cells in WI-38. In contrast, they showed high expression in some normal fibroblasts in eye tissue, providing another example of tissue-type SASP heterogeneity (Figure S6 and Figure 3B).

The number of cells with high expression of the selected five genes (i.e., *CDKN1A* (p21), *CDKN2A* (p16), *MIF*, *IGFBP7*, and *SERPINE2*) across PDL_50np in WI-38 human lung fibroblast cell line, fibroblasts in lung tissue, fibroblasts in skin tissue, and fibroblasts in eye tissue is illustrated in Figure 3A. *IGFBP7* was consistently upregulated in numerous senescent fibroblasts across all the groups analyzed. However, SASP transcriptional heterogeneity between tissues was also evident. For example, the number of senescent fibroblasts co-expressing *CDKN1A*, *IGFBP7*, and *SERPINE2* was larger in eye tissue than in other tissues.

*CDKN1A* (p21) was found to be overexpressed in WI-38 cells at PDL_50np, although it was reported that *CDKN1A* expression increased for 2 days following etoposide treatment but then decreased [17]. In the predicted senescent fibroblasts from lung, skin, and eye tissues, *CDKN1A* was elevated, reflecting the fact that senescent cells were identified by high SASP scores and p21^+^. It was reported that *CDKN1A* was generally overexpressed in senescent cells induced by multiple stimuli, i.e., replicative senescence (RS), ionizing radiation (IR), and etoposide (ETO) [17].

*CDKN2A* (p16) was overexpressed in WI-38 cells at PDL_50np, with reported increases in *CDKN2A* gene expression during etoposide treatment. It should be noted, however, that not all senescent cells exhibited high expression of *CDKN2A* (Figure S2), and the overall expression level in tissue samples was relatively low (Figure 3A). This makes *CDKN2A* a less efficient marker for identifying senescent cells within tissues.

The uniform manifold approximation and projection (UMAP) in Figure 3A presents the senescent cells annotated by high SASP scores and p21^+^, in which *IGFBP7* was highly expressed (see Table S1).

In Figure 3B, log-normalized counts of gene expression are shown for various SASP genes in normal fibroblasts and senescent/stressed fibroblasts obtained from WI-38 cells, as well as from lung, skin, and eye tissues. The pattern of overexpression seen in senescent cells for *IGFBP7* was similarly present for the genes encoding IGFBP5 and TIMP2. The overexpression of *IGFBP7*, with one-vs.-others comparisons, was also established in another scRNA-seq study on WI-38, with the amount of its overexpression compared to others being particularly large (specifically, RS: log2FC = 1.7, IR-induced senescent cells: log2FC = 1.3, and ETO-induced senescent cells: log2FC = 1.2) [17]. *IGFBP7* is associated with BRAF p.V600E-mediated senescence and apoptosis in BRAF p.V600E-positive human melanoma cell lines, and it was suggested that IGFBP7 could potentially serve as a more effective agent than RAF or MEK inhibitors [18]. In our analysis of the 10x single-cell RNA-seq platform, *IGFBP7* was the most consistent transcriptional SASP marker for detecting senescent fibroblasts from human lung, skin, and eye tissue samples.

The difference in SASP gene expression patterns between cell lines and tissues can be confirmed by many genes, including *SERPINE2*, *PAPPA*, *MMP1*, *MMP3*, and *CXCL8*. *SERPINE2* and *PAPPA* were clearly more highly expressed in WI-38 senescent fibroblasts but were silent in senescent fibroblasts from the three human tissues. *MMP1* was also clearly more expressed in WI-38 senescent fibroblasts, but it was silent in senescent fibroblasts in lung and skin tissues, and it was more highly expressed in normal fibroblasts in eye tissue. *MMP3* and *CXCL8* were silent in two PDL groups in WI-38, but they were more highly expressed in some normal fibroblasts in eye tissue. *SERPINE2* was identified as a marker for RS, IR-induced senescence, and ETO-induced senescence in single-cell data obtained from WI-38 [17]. *PAPPA* was identified as a replicative senescence marker in scRNA-seq data obtained from WI-38 [11]. However, these genes were not effective markers for identifying senescent cells due to their low expression levels in single-cell data obtained from lung, skin, and eye tissues.

The difference in SASP gene expression patterns between tissues can be observed with genes such as *CXCL12*, *SERPINE2*, *MMP1*, *MMP3*, and *CXCL8*. For instance, *CXCL12* showed higher expression in senescent/stressed fibroblasts from lung and skin tissues (Figures S4A and S5A) but was silent in eye tissue. In contrast, *SERPINE2*, *MMP1*, *MMP3*, and *CXCL8* were expressed at higher levels in some normal fibroblasts of the eye tissues but remained silent in other tissue fibroblasts.

Following the identification of the most confident control cells without p21/p16 expression and senescent cells expressing p21 or p16, we derived skin and eye tissue biomarkers that differentiated senescent fibroblasts from control fibroblasts, considering only genes expressed in at least 90% of cells in either population (see Figures S5C and S6C and Tables S2 and S3).

## 3. Discussion

We observed multiple layers of heterogeneity in SASP gene expression in WI-38: (1) A subset of SASP genes was highly expressed only in a subpopulation of mature senescent fibroblasts. (2) Another subset of SASP genes was highly expressed only in a subpopulation of earlier senescent fibroblasts. This motivated us to develop an ensemble of gene sets rather than relying on a single gene set. The ensemble of gene sets was applied to obtain fibroblast scores to predict senescent fibroblasts. We confirmed that SASP gene expression differed between single cells in the WI-38 cell line and fibroblasts in human lung tissue. This was an expected result, as multiple known factors can contribute to these differences, including disrupted cell–cell interactions, immortalization-associated alterations, cell line culture environment, selection pressures during extended culture, and genetic drift arising from replication errors. This is one of the reasons why we did not directly apply our cell line-derived ensemble of gene sets to predict senescent cells. Instead, we used it to identify the most confident normal and senescent cells, from which we derived new potential tissue-specific biomarkers for senescent cells.

To increase confidence in senescent cell detection, we selected 100 normal fibroblasts and 100 senescent/stressed fibroblasts such that the number of predicted senescent cells represented <1% of all fibroblasts in human lung and eye tissues and <2% in human skin tissue. These sample sizes were sufficient to identify biomarkers that distinguish normal from senescent fibroblasts across human tissues.

Our method was also applied to predict senescent fibroblasts in skin tissue and eye tissue, thereby allowing us to uncover another level of SASP heterogeneity, namely, tissue type heterogeneity. We believe that these computational experiments showcased the utility of our ensemble of gene sets based on cells with high expression of many SASP genes and other genes of interest.

To examine how the prediction of senescent cells depends on the number of selected cells, we repeated the analysis using different numbers of p21^−^/p16^−^ cells with the lowest SASP scores and p21^+^ or p16^+^ cells with the highest SASP scores (Figure S4D). The continuous decrease in *IGFBP7* mean expression within the senescent group indicated that the SASP score/EGS captured transcriptional signals associated with IGFBP7: cells with higher SASP scores tended to exhibit higher IGFBP7 expression. This trend supports our observation that *IGFBP7* transcription is the strongest transcriptional senescence biomarker in human lung, skin, and eye fibroblasts. The gradual decline in *IGFBP7* expression when expanding the senescent group suggests that *IGFBP7* may be involved across the progression from early senescence to mature senescence. Based on the model of the quiescence–senescence continuum [19], IGFBP7 may also contribute to the transitional states along this continuum.

Because p21 or p16 alone may not be sufficient to identify senescent cells, given that both can be expressed in quiescent cells, additional markers are needed to reduce false positives. p21-expressing quiescent cells (reversible cell-cycle arrest) do not exhibit strong SASP transcription. As cells deepen from shallow quiescence toward senescence, SASP gene expression progressively increases, which may correspond to increasing levels of *IGFBP7*. Some cells may re-enter the cell cycle, while others may continue into early senescence. We therefore propose that high *IGFBP7* expression among p21^+^ fibroblasts is a promising indicator of senescence in human tissue scRNA-seq datasets.

Pairing *IGFBP7* with p21^+^ expression provides evidence of SASP gene transcription and thus reduces false positives. Among the SASP genes considered for pairing with p21^+^ or p16^+^ expression, *IGFBP7* emerged as the most reliable, as it was consistently upregulated in senescent/stressed cells and readily detectable in scRNA-seq datasets from human tissues. In contrast, many other SASP factors were expressed at low or heterogeneous levels in single-cell data. For similar reasons, pairing *IGFBP7* with p16^+^ was less effective because *CDKN2A* (p16) expression was lower, heterogeneous, or less consistently detected in WI-38 senescent cells (Figure 1A). Altogether, we propose that the combination of p21^+^ and *IGFBP7* expression represents a strong candidate biomarker pair for identifying senescent fibroblasts at single-cell resolution.

Our approach to predict senescent fibroblasts in human tissue differed from a common way of studying molecular aging markers by comparing young-age and old-age samples, as our method began with the ensemble of gene sets derived from cell line data after considering SASP factor heterogeneity. The common method faces challenges due to the variable ratio of normal fibroblasts to senescent/stressed fibroblasts in age groups, which can be influenced by multiple factors. Fibroblasts in the young age group can have senescent fibroblasts, and fibroblasts in the old age group can also have normal fibroblasts. To address these concerns, our approach selected only more confident normal and senescent/stressed fibroblasts based on our gene set ensemble and utilized them to reduce the impact of the variable ratio of normal to senescent/stressed fibroblasts in a group.

IGFBP7 interacts with the extracellular domain of IGF1R in the absence of IGF1/2, resulting in the inhibition of the IRS1-PI3K-AKT signaling cascade [20] and consequently maintaining the normal activity of p21 and p27 within the nucleoplasm by avoiding their cytoplasmic retention mediated by AKT-triggered phosphorylation [21]. Oncogene-induced senescence due to IGFBP7 has also been implicated since it prompts activation of the p38 signaling pathway, leading to the stimulation of p53 and subsequent transcription of *CDKN1A* encoding for p21 [22]. A study revealed an association between IGFBP7 and the gene expression profiles related to cell cycle inhibitors, such as p21 and p27, in the context of thyroid carcinoma [23]. These findings collectively support the notion that elevated IGFBP7 levels are indicative of senescence, which is consistent with our observation that most senescent cells across various tissues exhibit higher expression of *IGFBP7*.

Cellular senescence signatures have great potential in identifying senescent cells, understanding aging processes, discovering biomarkers for senescence, and uncovering new therapeutic targets for both aging-related conditions and cancer. These signatures can also be applied to suggest tissue-specific variations in the magnitude and rate of senescence signal increments, as well as their diverse impact on tumors, which is also intricately tied to the specific tumor type. Developing new gene sets tailored for cancer research would be essential in understanding the intricate downstream mechanisms involved. Senescence can exert both positive and negative influences on tumorigenesis and tumor progression, making it crucial to disentangle these effects.

By analyzing time-resolved single-cell transcriptomic data from WI-38 human lung fibroblasts, we uncovered new layers of heterogeneity in SASP gene expression among senescent cells. Our findings demonstrate that SASP expression is not only temporally dynamic (distinguishing early from mature senescent states) but also varies across fibroblasts from different tissue origins. By constructing an ensemble of gene sets, we were able to robustly classify high-confidence SASP expression cells and normal cells, enabling more precise identification of senescence-associated biomarkers. This refined approach enhances our understanding of the diverse molecular programs active in senescence and provides a valuable resource for future research in aging and age-related diseases, including cancer, where SASP is known to influence tumor progression, immune responses, and tissue remodeling.

## 4. Materials and Methods

### 4.1. Fibroblast Single Cell Data

Senescence time-course scRNA-seq data for WI-38 human lung fibroblasts generated by the study of replicative senescence [11] was used to study SASP heterogeneity. This dataset consists of scRNA-seq data collected from multiple population doublings (PDLs). PDL 25 was used as a control, and it was compared to other PDLs (i.e., PDL 29, PDL 33, PDL 46, and PDL 50). We applied the proliferation score [24] to determine subgroups within each PDL. For example, PDL 25 cells were divided into two groups of PDL25np for non-proliferative cells and PDL25p for proliferative cells, and PDL 50 cells were divided into two groups of PDL_50np and PDL_50p. For our downstream analyses, we chose PDL_25p (900 cells), PDL_29np (573 cells), PDL_33np (657 cells), PDL_46np (1179 cells), and PDL_50np (991 cells) to ensure confident selection of normal fibroblasts in our control PDL 25 and senescent fibroblasts in other PDLs since the clearest feature of senescence is the cell cycle arrest (Figure S1).

### 4.2. Collection of SASP Factors and Other Genes of Interest

The gene names of the SASP factors were collected from multiple literature sources, including SenMayo’s list of 83 SASP factors [2]. The SenMayo gene set [2] contained 125 genes, comprising 83 SASP factors, 20 transmembrane genes, and 22 intracellular genes. A study on WI-38 single cells [17] mentioned SASP factors related to specific clusters.

For example, *COL1A1*, *MMP2*, and *CCL2* were associated with clusters 1 and 3, while *TIMP1*, *IGFBP7*, *MMP1*, and *SEPINE2* were linked to cluster 4. *IL6* was linked to cluster 5 during ETO-induced senescence. Another study on WI-38 replicative senescence [11] mentioned *PAPPA* as a prominent SASP factor. *IGF1*, *IGF2*, *IGF1R*, and *IGF2R* were also included as genes of interest since *PAPPA* may affect other cells with IGF receptors [25].

In addition to the above genes, cytokines and chemokines significantly altered by iron accumulation were added, which included *CSF3*, *CCL17*, *CCL19*, *CCL21*, and *CX3CL1*, since iron accumulation drives senescence and SASP [26]. *CDKN1A* (p21), *CDKN2A* (p16), and *TP53* were included to consider gene expression of these known genes related to cell cycle arrest. *SCAMP4* was added to the list of genes of interest since it was reported that SCAMP4 might accumulate on the surface of senescent cells and promote SASP factor secretion [27]. The total number of genes of interest was 106.

### 4.3. Constructing an Ensemble of Gene Sets for Representing Subpopulations

The mean values and standard deviations of genes across control cells of PDL_25p cells were computed and used to standardize the data, enabling calculation of gene-wise z-scores. To identify subpopulations defined by our genes of interest, we compared cells exhibiting overexpression of each gene of interest (z-score > 1.5) with the PDL_25p control cells (Figure 2A). The comparisons were performed for each PDL group using the FindMarkers() function in the Seurat R package (v4) [28], with parameters of *logfc.threshold* = 0.25 and *test.use* = “wilcox” when the number of cells in which a gene was overexpressed was greater than or equal to 50.

An auxiliary gene set named ‘lowSASP’ was also constructed for each PDL group, using cells in which SASP genes were either undetected or expressed at low levels. BRD4 inactivation via shRNA or BET inhibitors has been shown to repress SASP gene expression in oncogene-induced senescent cells [29], suggesting the possibility of senescence without the SASP [30]. Our lowSASP gene sets offer a valuable resource for detecting senescent cells with reduced or absent SASP gene expression. A total of 62 paired upregulated and downregulated gene sets were constructed.

### 4.4. Classification and Cross-Validation

To classify lung fibroblasts into two categories (i.e., normal fibroblasts and senescent/stressed fibroblasts) in human tissues, we defined a lung fibroblast senescence score for each gene set by calculating the mean z-score of upregulated genes minus the mean z-score of downregulated genes. Z-scores were computed using the mean and standard deviation of gene expression in PDL_25p cells, which were stored during the earlier stage of ensemble gene set construction.

The lung fibroblast senescence score for each cell was calculated by aggregating a vector of scores derived from the 62 gene set pairs. If the absolute maximum score in the vector was positive, the largest positive score in the vector was selected; if the absolute maximum score was negative, the smallest (most negative) score was chosen.

The distribution of the lung fibroblast senescence scores in our control cells (PDL_25p) showed slight overlap with the distribution of senescence scores in lung fibroblasts from the other PDL groups (PDL_29np, PDL_33np, PDL_46np, and PDL_50np). After applying a Gaussian approximation to these two distributions, we identified a threshold of 2.1 by minimizing the sum of false positives and false negatives. This threshold was then used for classification. Specifically, when a lung fibroblast senescence score exceeded this threshold, the cell was classified as senescent.

The overlap between the two distributions generated some misclassifications. However, these misclassifications might be linked to the ambiguous state of cells that exist in a transitional phase between normal cells and senescent cells. Moreover, it is possible that some cells at PDL_25p might be senescence-like cells or senescent cells, while some cells in other PDL groups might be normal-like cells or normal cells, although we did our best to choose more confident normal cells from PDL 25 and more confident senescent cells from other PDLs by using proliferation scores. To avoid protracted debates about the accuracy of the original labels of normal and senescent cells, we instead focused on the most confident cells by selecting the 100 cells with the lowest SASP scores and the 100 cells with the highest scores.

To evaluate the performance of the cell classification approach, we conducted three-fold cross-validation. We divided all cells into three folds for cross-validation. For each fold, we reconstructed an ensemble of gene sets from training cells and used them to classify the test cells into two groups. We then compared the resulting classification labels and the original labels assigned to the cells. Specifically, when focusing only on the 100 cells with the lowest SASP scores and the 100 cells with the highest SASP scores, our approach achieved perfect accuracy. This suggests that our score-based approach is a reliable way to distinguish between normal cells and senescent cells. By concentrating on obvious distinctions based on our scores, we could increase our confidence in identifying senescent cells. This level of high-confidence classification was essential for applying our method to the classification of fibroblasts in different tissues.

### 4.5. Prediction of Senescent Cells in Tissue

The ensemble of gene sets was built from scRNA-seq obtained from the WI-38 human lung fibroblast cell line, as described in previous sections. Here, we describe our approach to predicting senescent fibroblasts in tissues (e.g., lung tissue, skin tissue, and eye tissue). After computing z-scores for upregulated genes and downregulated genes for each gene set with stored mean values and standard deviations, we computed lung fibroblast senescence scores for each cell.

After selecting *n* cells with the lowest scores and *n* cells with the highest scores, we compared these two groups using the FindMarkers() function [28] with a parameter of *logfc.threshold* to identify upregulated and downregulated genes in senescent fibroblasts in a tissue. Following identification of a new gene set for senescent fibroblasts, we computed senescence scores for a tissue by the mean of z-scores of upregulated genes minus the mean of z-scores of downregulated genes.

Here, we describe how we determined the parameters of *n* and *logfc.threshold* and the classification threshold value. Two distinct distributions of the senescence scores for a tissue were expected. In order to obtain the most distinct distributions by using the most reasonable upregulated genes and downregulated genes in senescent cells, we optimized the parameters of *n* and *logfc.threshold*. Once we had the two most distinct distributions after the parameter optimization, the classification threshold value was determined by the middle of the mean values of the two distributions. When the senescence score for a fibroblast in a tissue was larger than this classification threshold value, it was classified as a senescent fibroblast in the tissue.

The scRNA-seq data utilized to compare lung, skin, and eye tissues included GSE132771, GSE157376, GSE159354, and GSE173896 for the lung tissue; GSE130973 for the skin tissue (all from donors over 50 years of age); and GSE157474 for the eye tissue (corneal limbus; donors aged 13, 53, 64, and 95 years). Only the fibroblasts annotated by the DISCO database were included in our data analyses.

We compared our SASP scores based on the ensemble of gene sets (SASP scores/EGS) derived from 62 upregulated and 62 downregulated gene sets, with the SenMayo module scores calculated using the AddModuleScore() function in the Seurat R package (v4) [28] and the 125 SenMayo genes [2] (Figure S4B). Batch correction using canonical correlation analysis (CCA) with ‘*project_id*’ as the integrated variable was performed prior to principal component analysis (PCA), UMAP, and clustering [28]. To further evaluate the predicted senescent cells, we assessed their proliferation status, as cell-cycle arrest is a key feature of senescence. The proliferation score, defined based on S-phase genes and G2/M cell-cycle genes [24], was used to identify proliferative cells, given that senescent cells exhibit G1-arrest.

We identified 119 cells with the highest SenMayo scores (top 1% among 11,889 fibroblasts in human lung tissue), among which 7 cells displayed nonzero proliferation scores, suggesting potential false positives. In contrast, all 119 cells with the highest SASP scores/EGS had zero proliferation score, strongly indicating that these cells were non-proliferative senescent cells. Moreover, an advantage of the SASP score/EGS is its ability to detect a larger number of senescent cell candidates, likely because the ensemble framework incorporates SASP gene expression heterogeneity across many gene sets. For instance, the UMAP showing senescent fibroblast predicted by SASP scores/EGS revealed additional senescent cell candidates in the lower-left region (Figure S4C).

## 5. Conclusions

Our study provides a comprehensive framework for understanding the complexity of temporal SASP gene expression in senescent fibroblasts. By leveraging single-cell RNA sequencing data from WI-38 human lung fibroblasts, we uncovered both temporal and subpopulation-specific heterogeneity in SASP expression. The development of an ensemble of gene sets allowed us to robustly identify senescent or other stressed cells across diverse tissues, revealing *IGFBP7* as a consistently upregulated marker in p21^+^ fibroblast subpopulations. Importantly, our approach moves beyond traditional age-based comparisons by focusing on confidently classified senescent and normal cells, enabling more precise tissue-specific biomarker discovery. The observed tissue-specific variations in SASP profiles further highlight the need for context-aware strategies in aging and cancer research. This work lays the groundwork for improved detection of senescent cells and opens new avenues for exploring how senescent cells contribute to tissue aging and disease progression.

## Data Availability

This study did not generate new datasets. All analyses were conducted using publicly available data, as detailed in Section 4. Additional Supplementary Materials associated with this work are available at https://github.com/hyunsoo77/SASP_score (accessed on 10 March 2026).

## Supplementary Materials

The following supporting information can be downloaded at: https://www.mdpi.com/article/10.3390/ijms27073012/s1.
