# Supplementary material for: Ensemble of Time-Evolving SASP Gene Sets Identifies IGFBP7 and CDKN1A as a Potential Marker Pair for Senescent Fibroblast Subpopulations Across Tissues
Source: Int J Mol Sci. 2026 Mar 26;27(7):3012. doi: 10.3390/ijms27073012 (PMC13073673; doi:10.3390/ijms27073012)
Supplement: Supplementary file 1 [file ijms-27-03012-s001.zip › supplementary_figures HK.pdf]

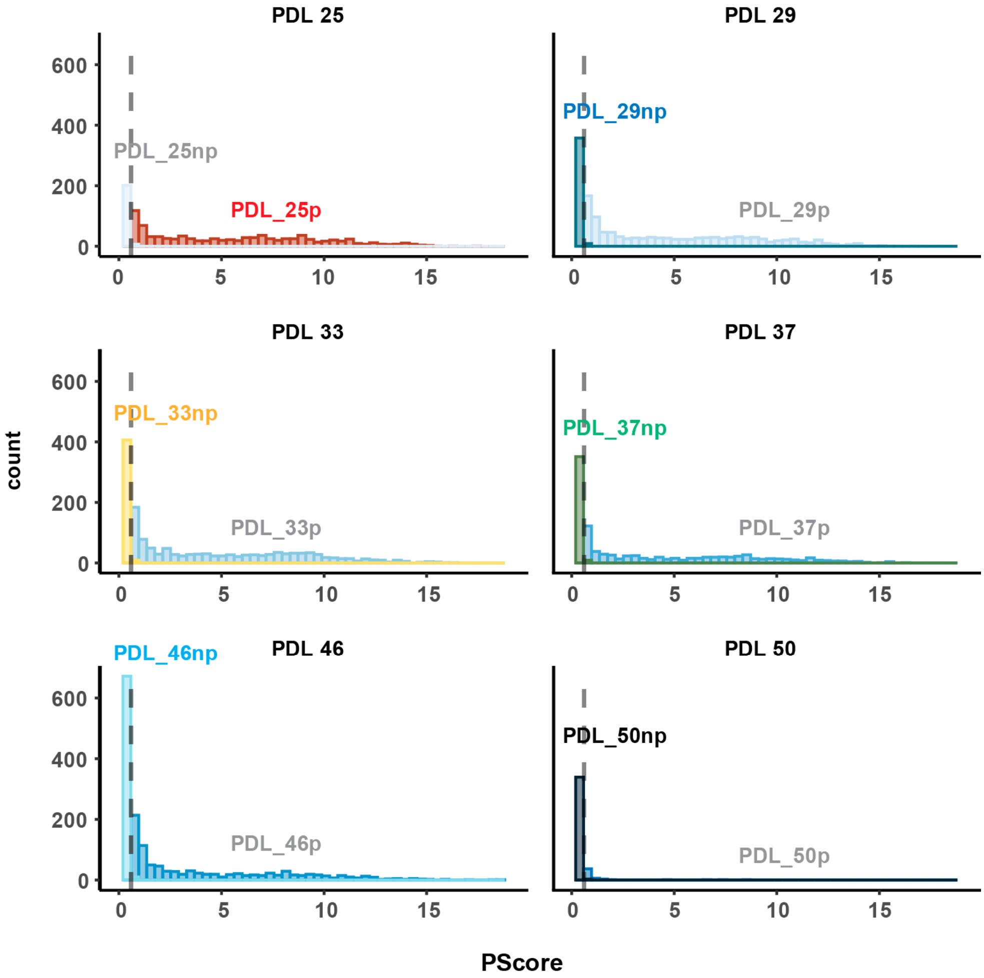

Figure S1. Histogram of proliferation scores for the population doubling (PDL) groups. Each PDL group was divided into two subgroups: nonproliferating (NP) and proliferating (P) groups, based on a threshold value of 0.6. This threshold was determined to ensure that 95% of PDL 50 cells were non-proliferative, as SA-β-gal-positive cells comprised approximately 95% of all cells at PDL 50. To obtain more confidently distinguishable normal fibroblasts and senescent fibroblasts, we selected the following subgroups: PDL_25p for normal fibroblasts and PDL_29np, PDL_33np, PDL_37np, PDL_46np, and PDL_50np for senescent fibroblasts, as cell cycle arrest is a key characteristic of senescent cells.

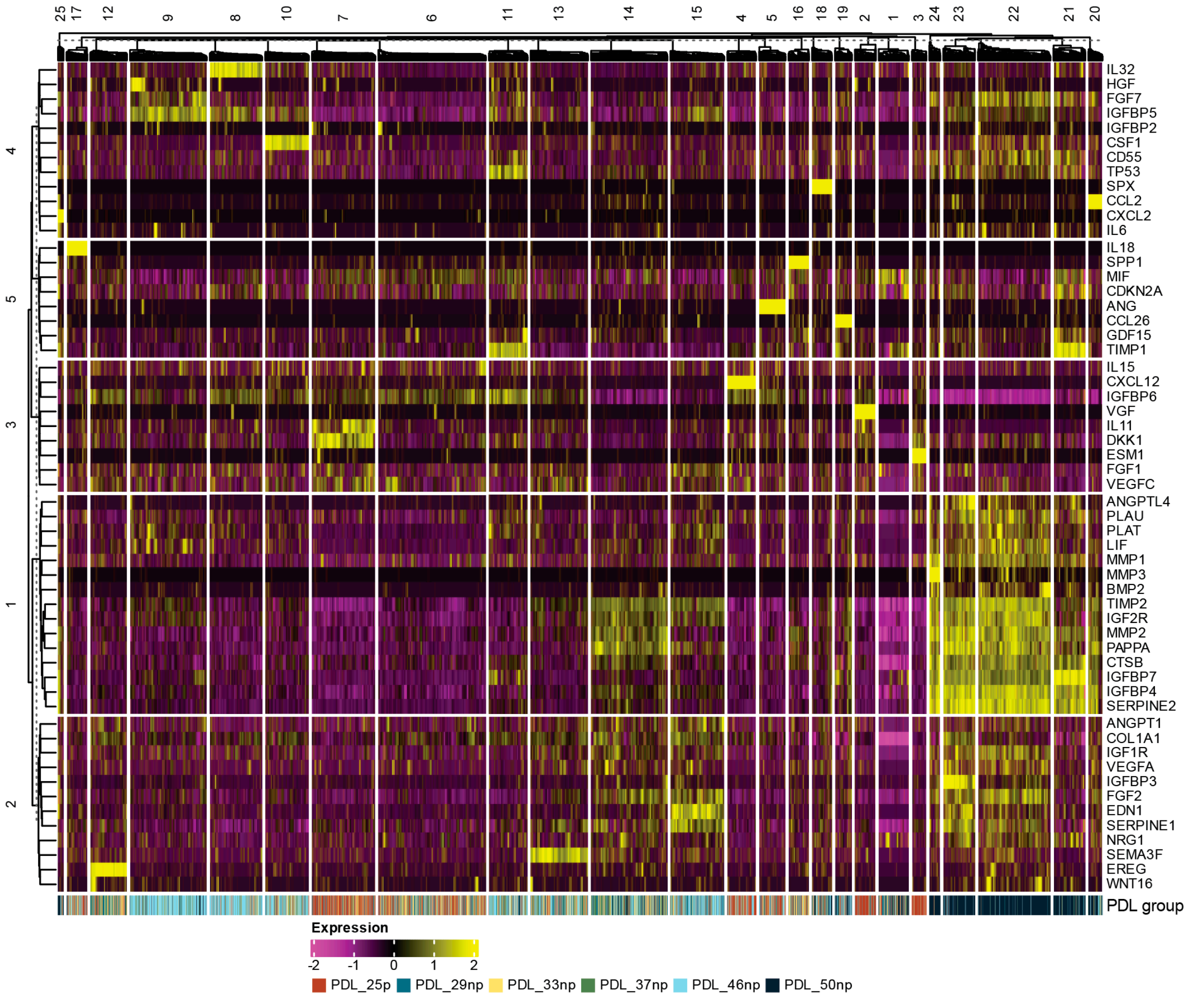

Figure S2. Heatmap of normalized gene expression values of SASP factors and other genes of interest in the WI-38 human lung fibroblast cell line. A selection of SASP factors, along with additional genes including *CDKN1A* (p21), *CDKN2A* (p16), *TP53*, *IGF1R*, and *IGF2R*, were summarized by five-row (gene) clustering for visualization purposes. Row cluster 1 exhibited high expression levels of *IGFBP7*, *IGFBP4*, *PAPPA*, *IGF2R*, *TIMP2*, *MMP2*, *CTSB*, and *SERPINE2.* All normal fibroblasts (PDL_25p) as well as earlier senescent fibroblasts (PDL_29np, PDL_33_np, PDL_37np, and PDL_46np), and mature senescent fibroblasts (PDL_50np) were summarized by 25 column (cell) clustering for visualization purposes. Column clusters 22 and 23 displayed higher expression levels of *PAPPA* and *IGF2R*. Column cluster 23 showed increased expression of *IGFBP3*.

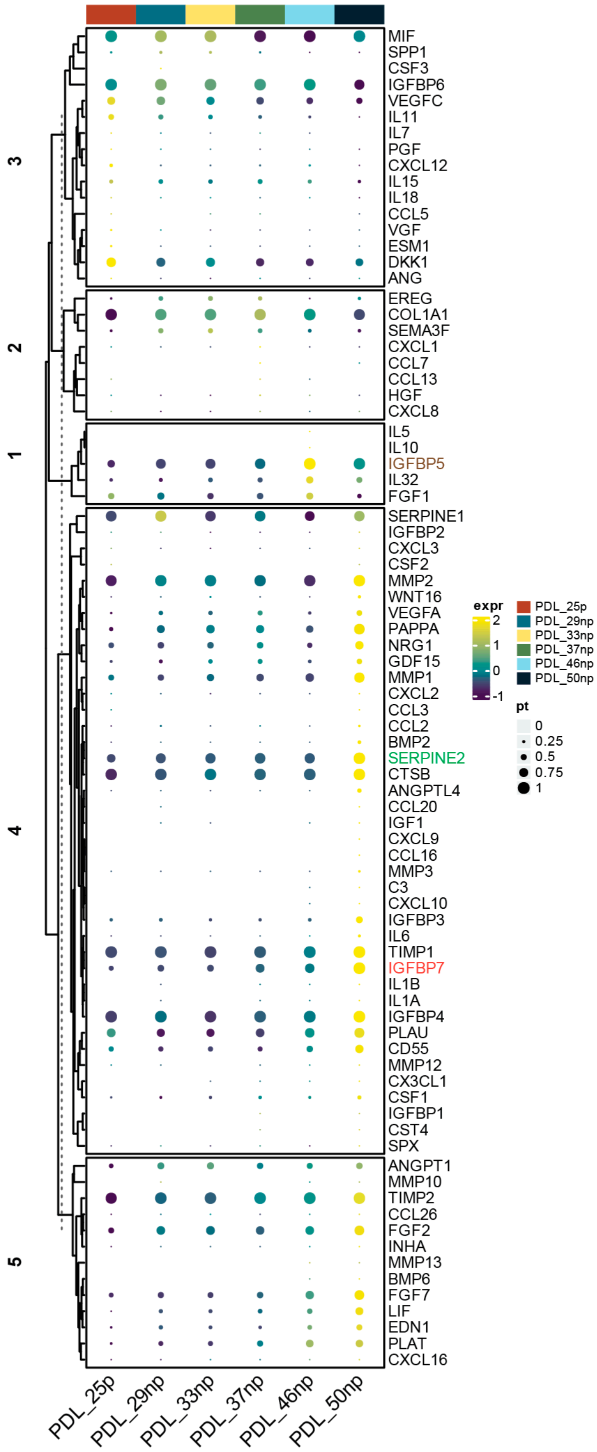

Figure S3. Dot plot of gene expression of SASP factors in WI-38 lung fibroblast cell line. The expression values for a group represent the mean values of normalized expression values across cells within a group. The size of dots corresponds to the percentage of cells where gene expression is nonzero within each group. Row cluster 3 revealed that some genes were highly expressed in normal fibroblasts. Row cluster 1 showed that *IGFBP5* exhibited high expression in PDL_46np cells. Meanwhile, row clusters 4 and 5 indicated that *IGFBP7, TIMP2, TIMP1, MMP1, MMP2, CTSB,* and *SERPINE2* were highly expressed in the mature senescent cells.

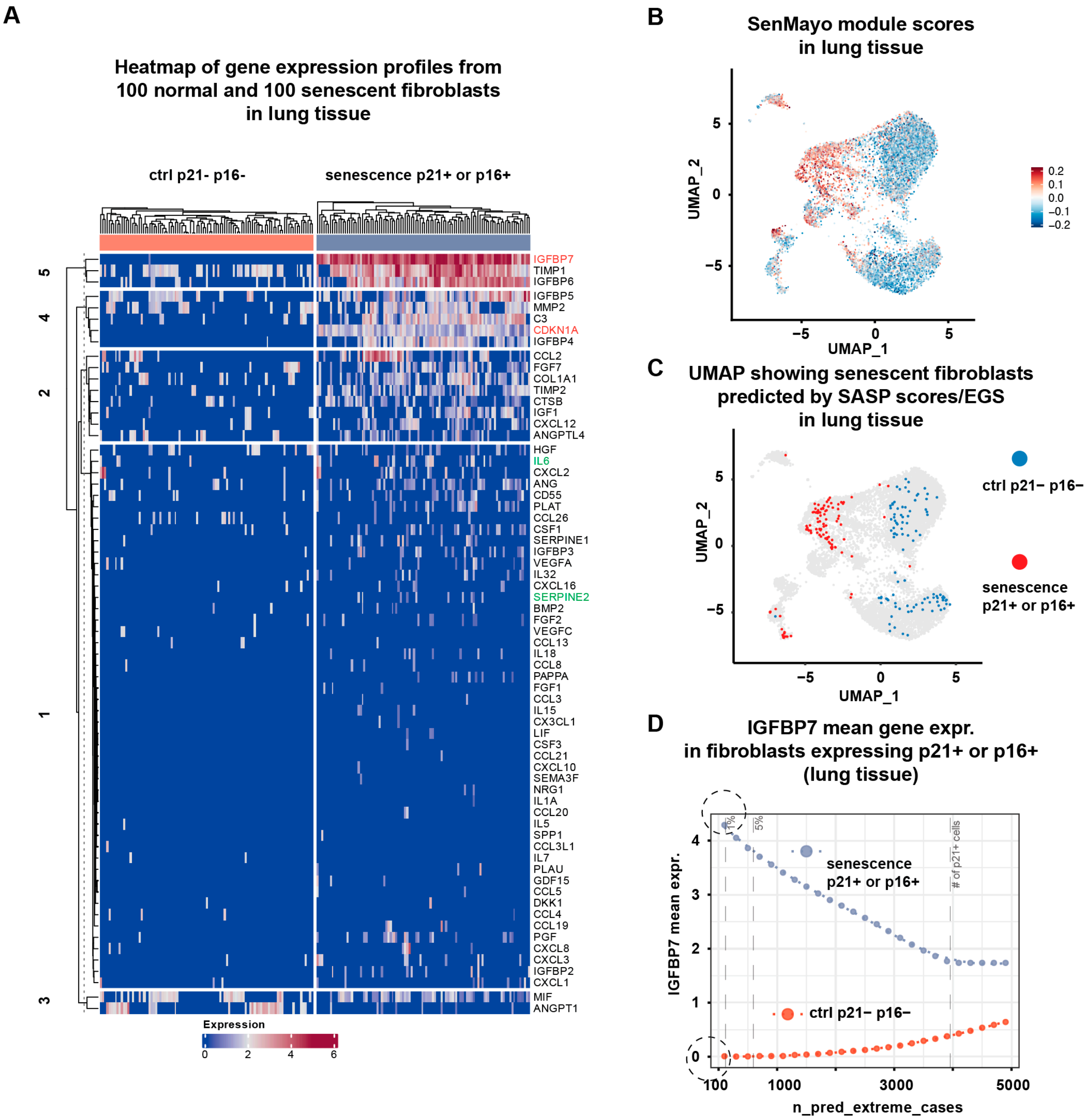

Figure S4. (**A**) Heatmap of absolute gene expression in fibroblasts from human lung tissue. SASP-related genes and additional genes of interest were clustered, and row cluster 5 included *IGFBP7*, *TIMP1*, and *IGFBP6*. The heatmap columns represent 100 normal fibroblasts and 100 senescent/stressed fibroblasts predicted by SASP scores based on an ensemble of gene sets (SASP scores/EGS). High expression of *IGFBP7* and *CDKN1A* was enriched in the 100 predicted senescent/stressed fibroblasts. (**B**) SenMayo module scores for fibroblasts in human lung tissue, calculated using the 125-gene SenMayo signature. (**C**) UMAP showing senescent fibroblasts predicted by SASP scores/EGS in lung tissue. This panel corresponds to the same dataset shown in Figure 3A, but it is presented here to facilitate direct comparison with the SenMayo module scores in Figure S4B. (**D**) IGFBP7 mean gene expression in fibroblasts expressing p21^{+} or p16^{+} in lung tissue.

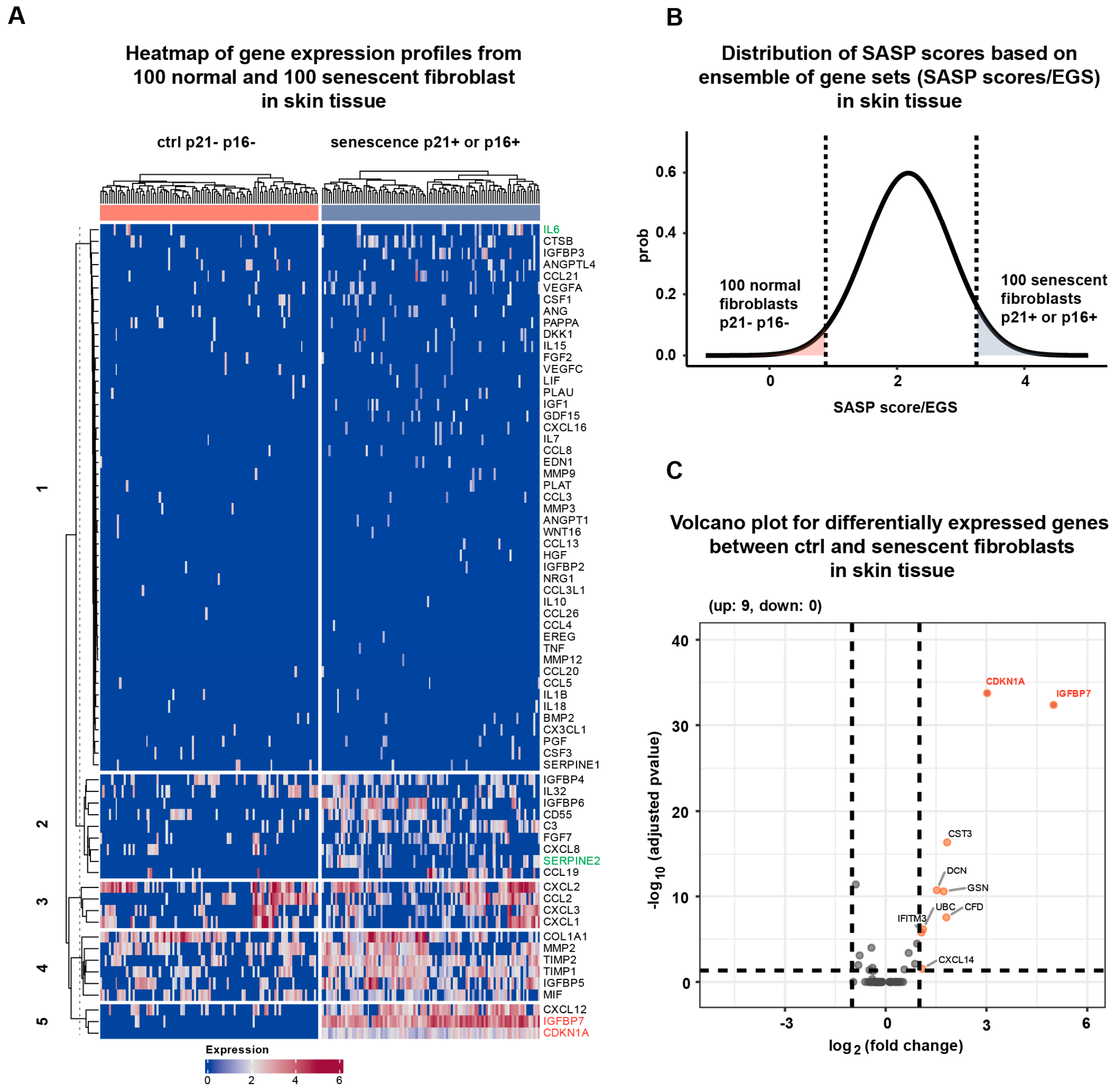

Figure S5. (**A**) Heatmap of absolute gene expression in fibroblasts from human skin tissue. SASP-related genes and additional genes of interest were clustered, and row cluster 5 included *IGFBP7*, *CXCL12*, and *CDKN1A*. The heatmap columns represent 100 normal fibroblasts and 100 senescent/stressed fibroblasts predicted by SASP scores/EGS. High expression of *IGFBP7* and *CDKN1A* was enriched among the 100 senescent/stressed fibroblasts. (**B**) Distribution of SASP scores/EGS in skin tissue. (**C**) Volcano plot showing differentially expressed genes between control and senescent fibroblasts in skin tissue.

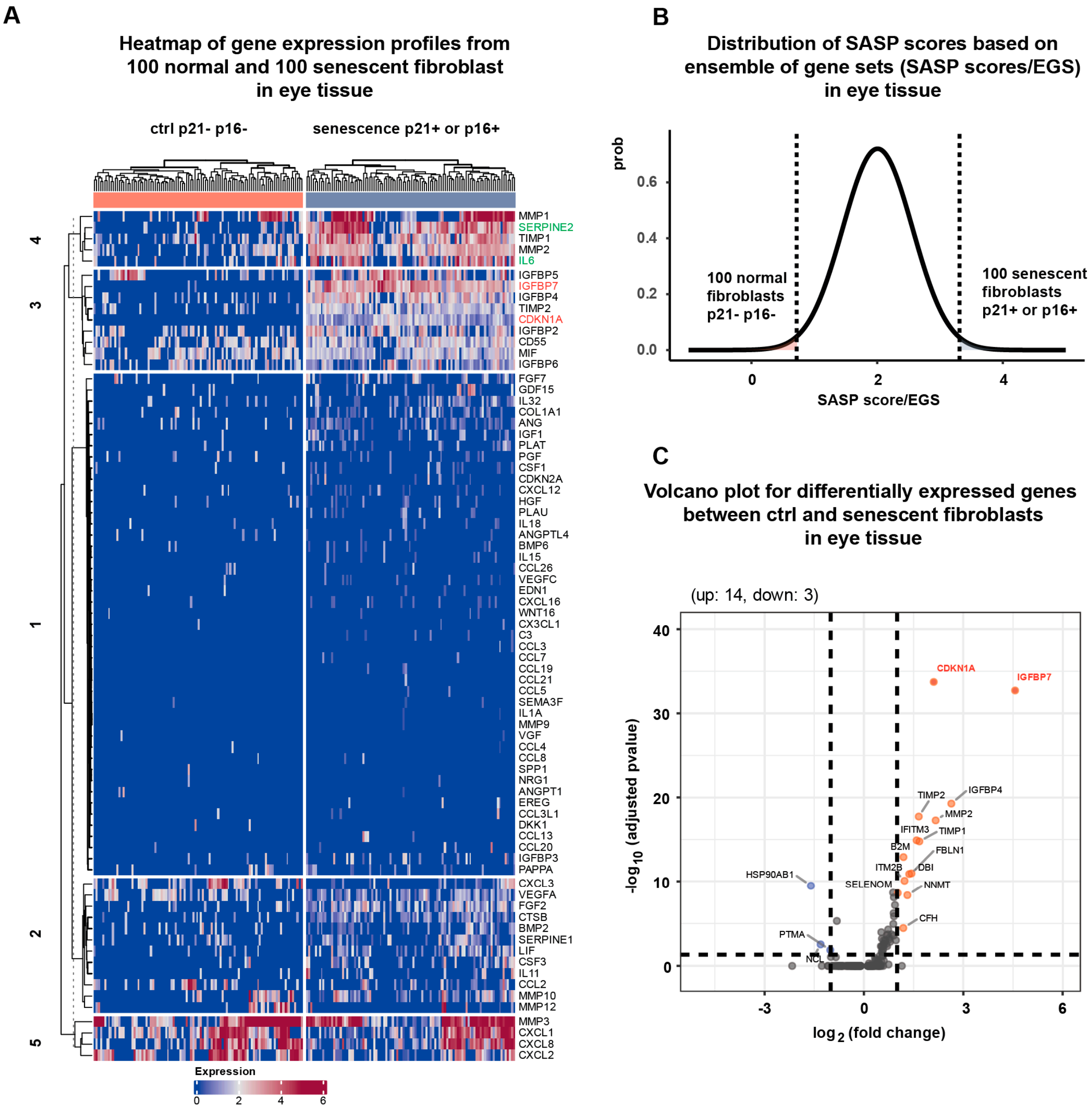

Figure S6. (**A**) Heatmap of absolute gene expression in fibroblasts from human eye tissue. SASP-related genes and additional genes of interest were clustered, and row cluster 3 included *IGFBP7*, *IGFBP2/4/5/6*, *SERPINE2*, *TIMP2*, *CD55*, *MIF*, and *CDKN1A*. The heatmap columns represent 100 normal fibroblasts and 100 senescent/stressed fibroblasts predicted by SASP scores/EGS. The 100 predicted senescent/stressed fibroblasts showed higher expression of *IGFBP7* and *CDKN1A*. (**B**) Distribution of SASP scores/EGS in eye tissue. (**C**) Volcano plot showing differentially expressed genes between control and senescent fibroblasts in eye tissue.
