# Supplementary material for: Ensemble of Time-Evolving SASP Gene Sets Identifies IGFBP7 and CDKN1A as a Potential Marker Pair for Senescent Fibroblast Subpopulations Across Tissues
Source: Int J Mol Sci. 2026 Mar 26;27(7):3012. doi: 10.3390/ijms27073012 (PMC13073673; doi:10.3390/ijms27073012)
Supplement: Supplementary file 1 [file ijms-27-03012-s001.zip › supplementary_fig_s4.pdf]

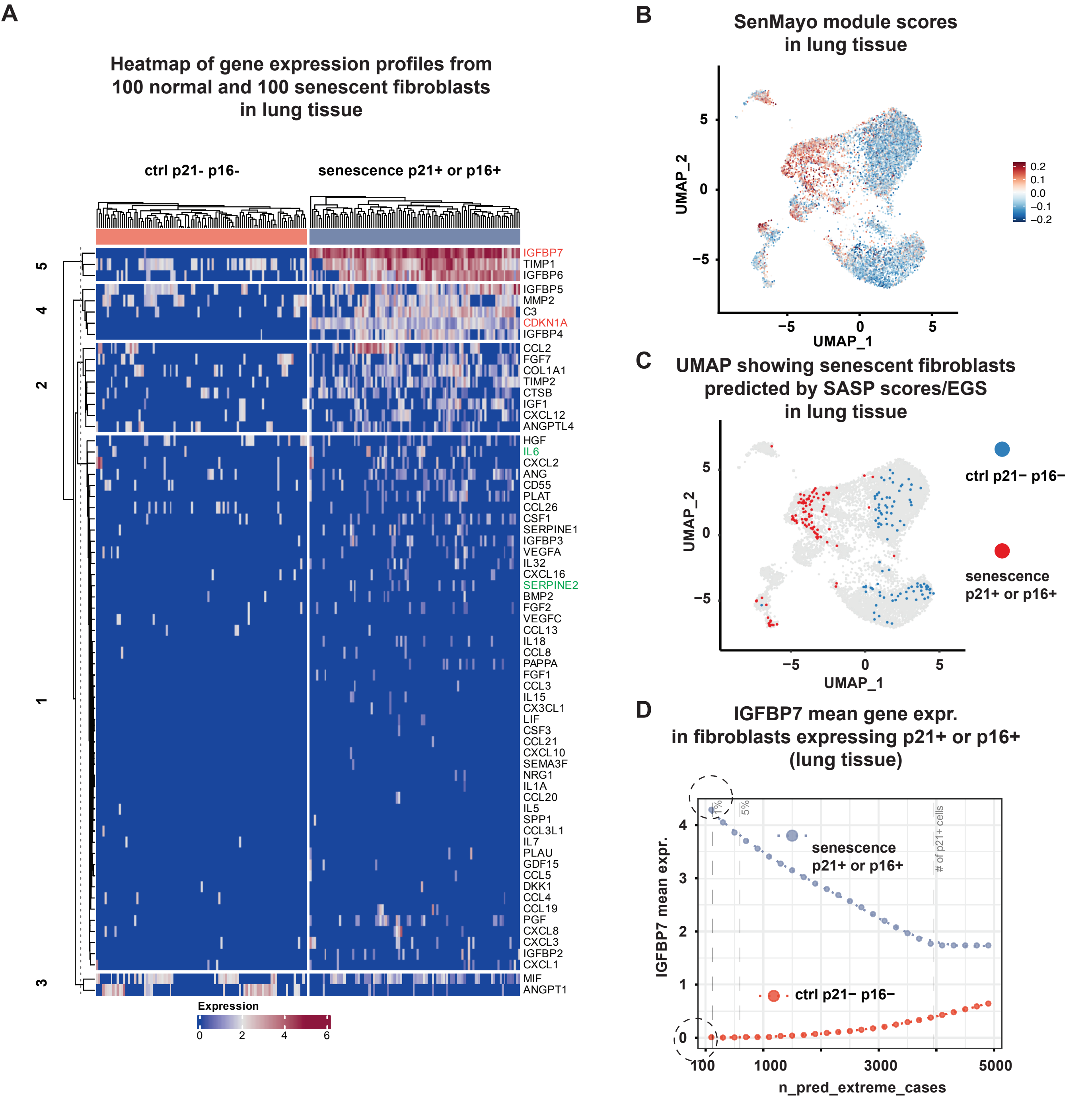
A
Heatmap of gene expression profiles from 100 normal and 100 senescent fibroblasts in lung tissue
ctrl p21- p16-
senescence p21+ or p16+
5
4
2
1
3
IGFBP7
TIMP1
IGFBP6
IGFBP5
MMP2
C3
CDKN1A
IGFBP4
CCL2
FGF7
COL1A1
TIMP2
CTSB
IGF1
CXCL12
ANGPTL4
HGF
IL6
CXCL2
ANG
CD55
PLAT
CCL26
CSF1
SERPINE1
IGFBP3
VEGFA
IL32
CXCL16
SERPINE2
BMP2
FGF2
VEGFC
CCL13
IL18
CCL8
PAPPA
FGF1
CCL3
IL15
CX3CL1
LIF
CSF3
CCL21
CXCL10
SEMA3F
NRG1
IL1A
CCL20
IL5
SPP1
CCL3L1
IL7
PLAU
GDF15
CCL5
DKK1
CCL4
CCL19
PGF
CXCL8
CXCL3
IGFBP2
CXCL1
MIF
ANGPT1
Expression
0
2
4
6
B
SenMayo module scores in lung tissue
UMAP_2
UMAP_1
5
0
−5
0.2
0.1
0.0
−0.1
−0.2
C
UMAP showing senescent fibroblasts predicted by SASP scores/EGS in lung tissue
UMAP_2
UMAP_1
ctrl p21− p16−
senescence p21+ or p16+
D
IGFBP7 mean gene expr. in fibroblasts expressing p21+ or p16+ (lung tissue)
IGFBP7 mean expr.
n_pred_extreme_cases
1%
5%
# of p21+ cells
senescence p21+ or p16+
ctrl p21− p16−
0
1
2
3
4
100
1000
3000
5000
