# Supplementary material for: Ensemble of Time-Evolving SASP Gene Sets Identifies IGFBP7 and CDKN1A as a Potential Marker Pair for Senescent Fibroblast Subpopulations Across Tissues
Source: Int J Mol Sci. 2026 Mar 26;27(7):3012. doi: 10.3390/ijms27073012 (PMC13073673; doi:10.3390/ijms27073012)
Supplement: Supplementary file 1 [file ijms-27-03012-s001.zip › supplementary_fig_s5.pdf]

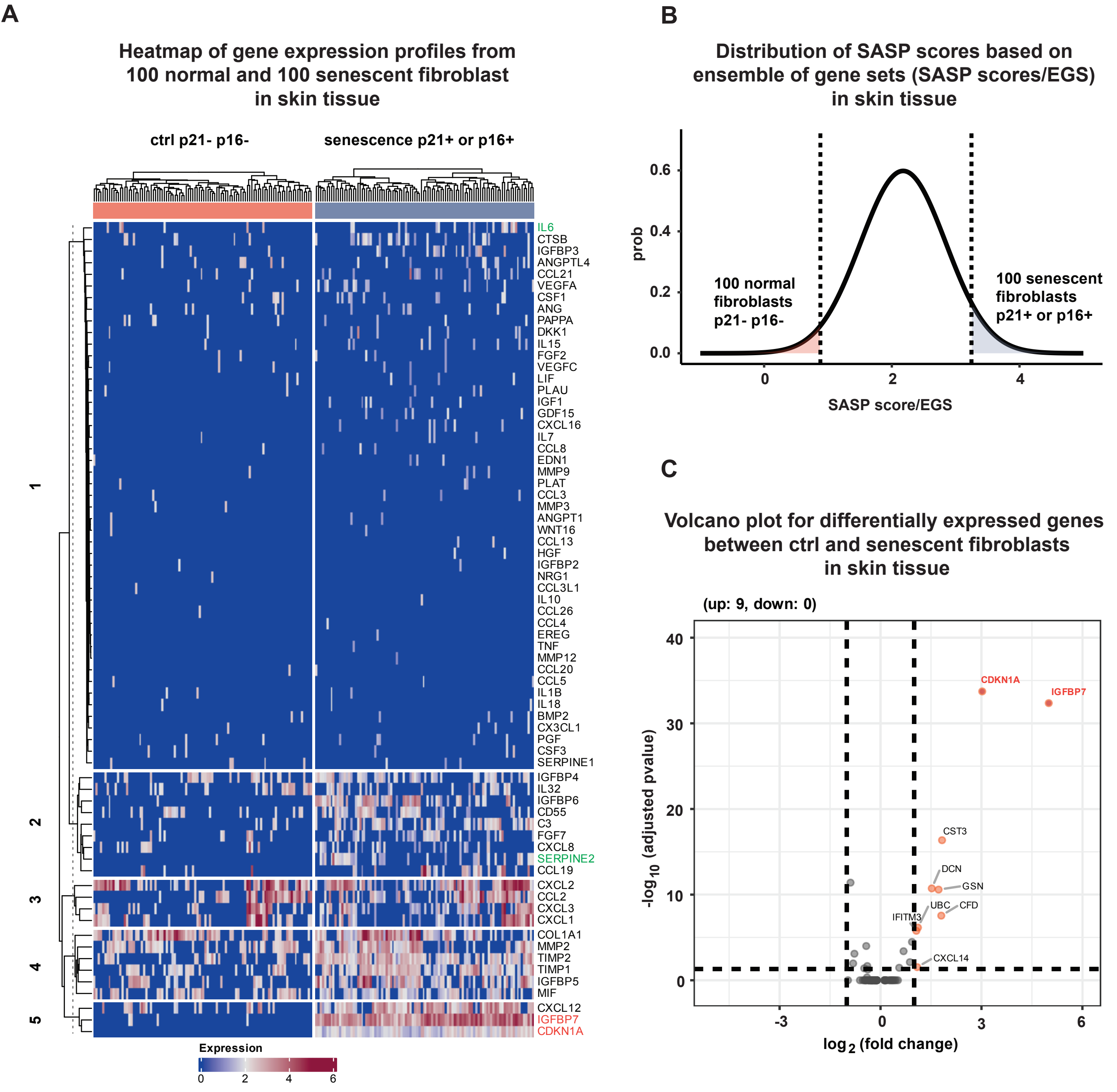
A
Heatmap of gene expression profiles from 100 normal and 100 senescent fibroblast in skin tissue
ctrl p21- p16-
senescence p21+ or p16+
1
2
3
4
5
IL6
CTSB
IGFBP3
ANGPTL4
CCL21
VEGFA
CSF1
ANG
PAPPA
DKK1
IL15
FGF2
VEGFC
LIF
PLAU
IGF1
GDF15
CXCL16
IL7
CCL8
EDN1
MMP9
PLAT
CCL3
MMP3
ANGPT1
WNT16
CCL13
HGF
IGFBP2
NRG1
CCL3L1
IL10
CCL26
CCL4
EREG
TNF
MMP12
CCL20
CCL5
IL1B
IL18
BMP2
CX3CL1
PGF
CSF3
SERPINE1
IGFBP4
IL32
IGFBP6
CD55
C3
FGF7
CXCL8
SERPINE2
CCL19
CXCL2
CCL2
CXCL3
CXCL1
COL1A1
MMP2
TIMP2
TIMP1
IGFBP5
MIF
CXCL12
IGFBP7
CDKN1A
Expression
0
2
4
6
B
Distribution of SASP scores based on ensemble of gene sets (SASP scores/EGS) in skin tissue
prob
0.6
0.4
0.2
0.0
100 normal fibroblasts p21- p16-
100 senescent fibroblasts p21+ or p16+
0
2
4
SASP score/EGS
C
Volcano plot for differentially expressed genes between ctrl and senescent fibroblasts in skin tissue
(up: 9, down: 0)
40
30
20
10
0
-log10 (adjusted pvalue)
CDKN1A
IGFBP7
CST3
DCN
GSN
UBC
CFD
IFITM3
CXCL14
-3
0
3
6
log2 (fold change)
