# Supplementary material for: Ensemble of Time-Evolving SASP Gene Sets Identifies IGFBP7 and CDKN1A as a Potential Marker Pair for Senescent Fibroblast Subpopulations Across Tissues
Source: Int J Mol Sci. 2026 Mar 26;27(7):3012. doi: 10.3390/ijms27073012 (PMC13073673; doi:10.3390/ijms27073012)
Supplement: Supplementary file 1 [file ijms-27-03012-s001.zip › supplementary_fig_s6.pdf]

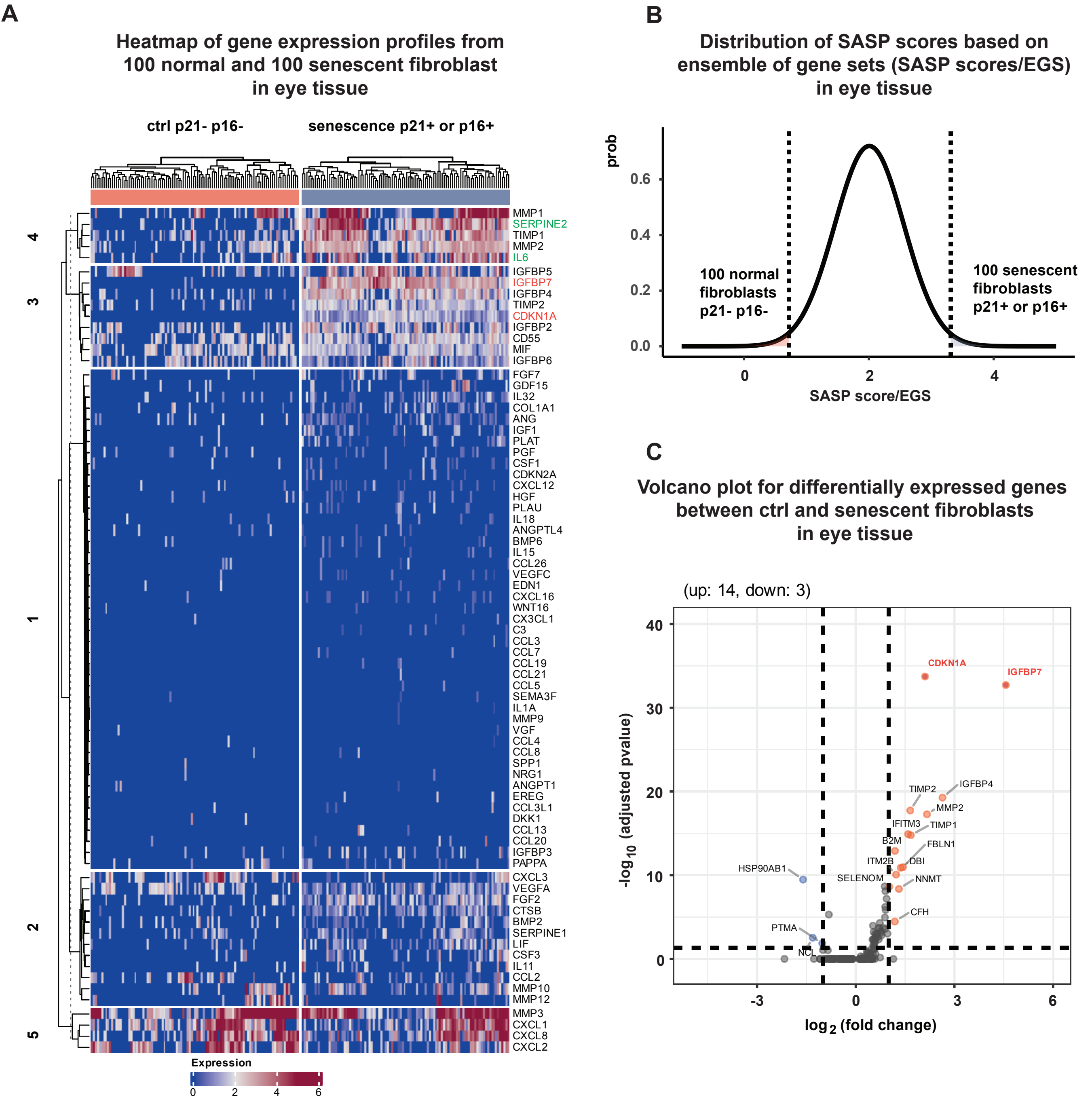
A
Heatmap of gene expression profiles from 100 normal and 100 senescent fibroblast in eye tissue
ctrl p21- p16-
senescence p21+ or p16+
MMP1
SERPINE2
TIMP1
MMP2
IL6
IGFBP5
IGFBP7
IGFBP4
TIMP2
CDKN1A
IGFBP2
CD55
MIF
IGFBP6
FGF7
GDF15
IL32
COL1A1
ANG
IGF1
PLAT
PGF
CSF1
CDKN2A
CXCL12
HGF
PLAU
IL18
ANGPTL4
BMP6
IL15
CCL26
VEGFC
EDN1
CXCL16
WNT16
CX3CL1
C3
CCL3
CCL7
CCL19
CCL21
CCL5
SEMA3F
IL1A
MMP9
VGF
CCL4
CCL8
SPP1
NRG1
ANGPT1
EREG
CCL3L1
DKK1
CCL13
CCL20
IGFBP3
PAPPA
CXCL3
VEGFA
FGF2
CTSB
BMP2
SERPINE1
LIF
CSF3
IL11
CCL2
MMP10
MMP12
MMP3
CXCL1
CXCL8
CXCL2
4
3
1
2
5
Expression
0
2
4
6
B
Distribution of SASP scores based on ensemble of gene sets (SASP scores/EGS) in eye tissue
prob
0.6
0.4
0.2
0.0
100 normal fibroblasts p21- p16-
100 senescent fibroblasts p21+ or p16+
0
2
4
SASP score/EGS
C
Volcano plot for differentially expressed genes between ctrl and senescent fibroblasts in eye tissue
(up: 14, down: 3)
40
30
20
10
0
-log10 (adjusted pvalue)
CDKN1A
IGFBP7
IGFBP4
TIMP2
MMP2
IFITM3
TIMP1
B2M
FBLN1
ITM2B
DBI
HSP90AB1
SELENOM
NNMT
CFH
PTMA
NCL
-3
0
3
6
log2 (fold change)
