# Supplementary figures and images for: Ensemble of Time-Evolving SASP Gene Sets Identifies IGFBP7 and CDKN1A as a Potential Marker Pair for Senescent Fibroblast Subpopulations Across Tissues
Source: Int J Mol Sci. 2026 Mar 26;27(7):3012. doi: 10.3390/ijms27073012 (PMC13073673; doi:10.3390/ijms27073012)

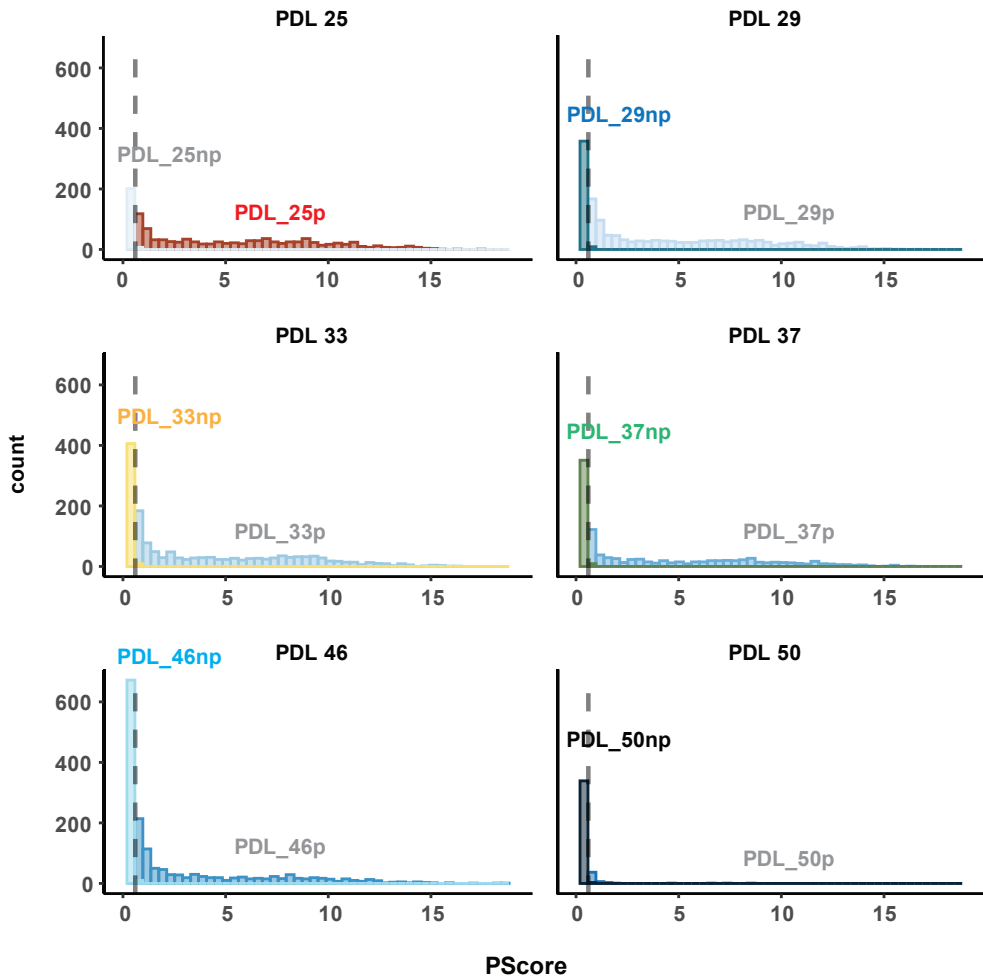

Supplement: Supplementary file 1 [file ijms-27-03012-s001.zip › supplementary_fig_s1.pdf]

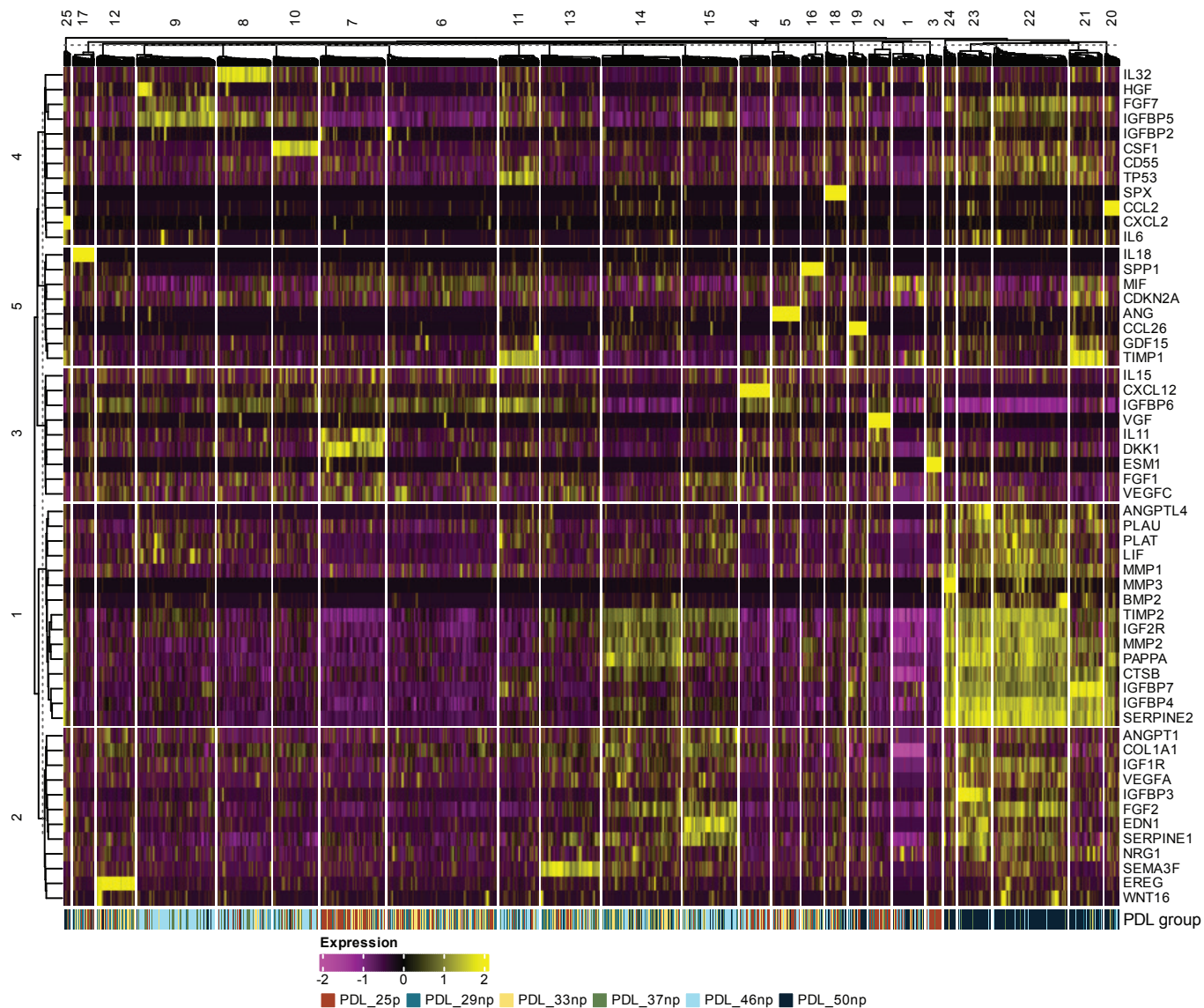

Supplement: Supplementary file 1 [file ijms-27-03012-s001.zip › supplementary_fig_s2.pdf]

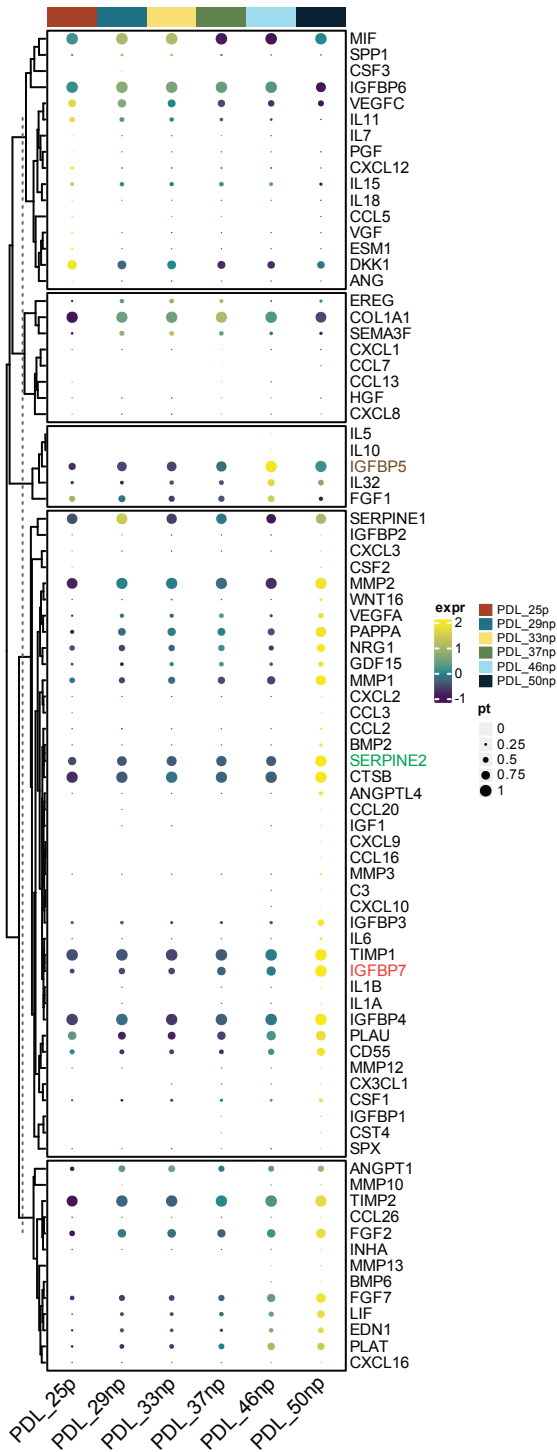

Supplement: Supplementary file 1 [file ijms-27-03012-s001.zip › supplementary_fig_s3.pdf]
